# Mechanical characterizations of braided composite stents made of helical polyethylene terephthalate strips and NiTi wires

**DOI:** 10.1515/ntrev-2019-0016

**Published:** 2019-11-06

**Authors:** Qingli Zheng, Pengfei Dong, Zhiqiang Li, Xinwei Han, Changchun Zhou, Meiwen An, Linxia Gu

**Affiliations:** Institute of Biomedical Engineering, College of Biomedical Engineering, Taiyuan University of Technology, Taiyuan 030024, China; Department of Mechanical & Materials Engineering, University of Nebraska, Lincoln, NE 68588, United States of America; Institute of Applied Mechanics, College of Mechanical and Vehicle Engineering Taiyuan University of Technology, Taiyuan 030024, China; Department of Interventional Radiology, The First Affiliated Hospital of Zhengzhou University, Henan, China; National Engineering Research Center for Biomaterials, Sichuan University, 610064, Chengdu, China; Institute of Biomedical Engineering, College of Biomedical Engineering, Taiyuan University of Technology, Taiyuan 030024, China; Department of Biomedical and Chemical Engineering and Sciences, Florida Institute of Technology, Melbourne, FL 32901-6975, United States of America

**Keywords:** Braided composite stent, wire interaction, radial strength, flexibility, finite element method

## Abstract

The novel braided composite stent (BCS), woven with both nitinol wires and polyethylene terephthalate (PET) strips, were characterized and compared with the braided nitinol stent in the same weaving pattern. Finite element models simulating the stent compression and bending were developed to quantify its radial strength and longitudinal flexibility. The interaction between the nitinol wires and the PET strips were also delineated. Results showed that the PET strips enforced more constrains on the BCS and thus enhance its radial strength especially at a larger compression load. The longitudinal flexibility of the BCS was less sensitive to the presence of the PET strips. This work suggested that the novel design of the BCS could acquire the advantage of a covered stent without compromising its mechanical performance. The fundamental understanding of the braided composite stent will facilitate a better device design.

## 1 Introduction

Braided nitinol stents (BNS) are increasingly popular in treating arterial occlusions, especially peripheral arterial disease [1]. Compared to the laser-cut stents, the BNS tend to have a reduced radial strength and better flexibility [2]. The radial strength of the stent was essential to keep the narrowed vessel open [3]. The flexibility of the stent was desired to move along or scaffold a curved vessel. Despite the demonstrated acute outcomes of a stented vessel, the long-term patency of the BNS remains a challenge due to tissue ingrowth through the mesh structure of stents [4].

Covered stents, *i.e.*, stents covered by a fabric or graft material such as polytetrafluoroethylene or polyethylene terephthalate (PET), were designed to mitigate the tissue ingrowth as well as to seal the vessel perforation [5]. However, compared to a bare metallic stent, the covered stent is harder to maneuver through tortuous anatomy without the risk of kinking and migration [6, 7]. This led to undesired vessel patency as well as tissue damage and increase incidence of limb thrombosis [8]. Recently a braided composite stent (BCS) woven using PET strips and nitinol (NiTi) wires was proposed to improve the radial strength as well as the flexibility of a BNS [9]. Considering the minimal porosity of the BCS, it drove us to consider the BCS as a potential alternative to the covered stent.

Finite element method (FEM) has proven to be an effective tool for the characterization of the stent performance and its underlying mechanism. It could visualize the mechanics virtually anywhere in the computational domain, a feature that is not affected by experimental techniques. Kim *et al.* developed a 3D model to quantify the mechanical behavior of braided stents validated by experiments [10]. The FEM results from Zhao *et al.* showed that the braided stent had a relative small radial strength than the welded or laser cut ones [2]. De Beule *et al.* adopted a virtual optimization algorithm to design a braided stent with less foreshortening while maintaining the radial stiffness [11]. However, no FEM studies were related to the BCS.

In this work, the mechanical behaviors of the BCS were investigated through FEM. Three-dimensional models of the BCS and the corresponding BNS in the same weaving pattern were developed to evaluate and compare their radial strength and flexibility. The BCS model was validated by the published experimental results [9]. The effects of PET strips as well as the interaction between PET strips and NiTi wires were delineated. Quantitative evaluation of the mechanical performance of the BCS and its underlying mechanisms could facilitate an optimal design of the stent.

## 2 Materials and methods

**Figure 1:**
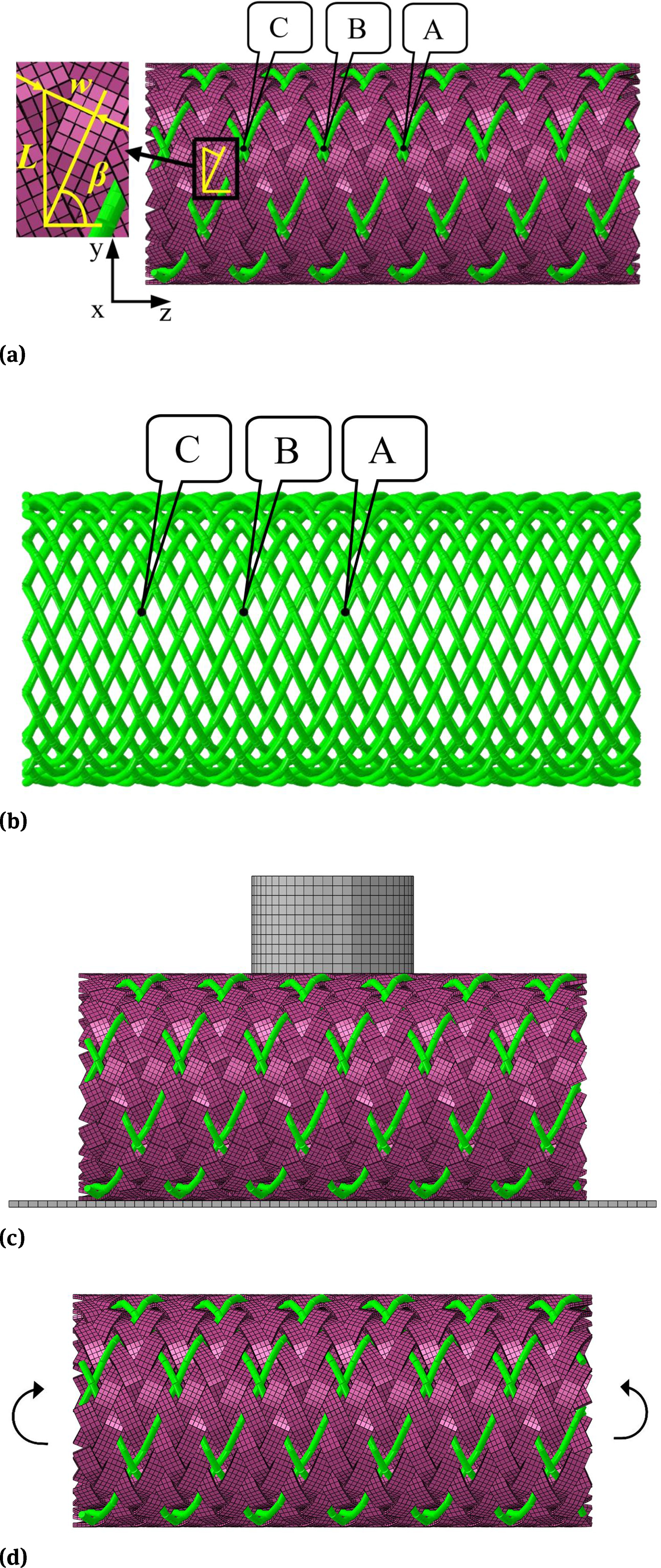
Three-dimensional models of (a) braided composite stent and (b) braided nitinol stents, (c) compression test, (d) bending test.

Three-dimensional models of the BCS as well as its counterpart BNS were constructed as shown in Figure 1. Both BCS and BNS have an outer diameter of 7 mm, length of 15mm, and a braided angle *β* as 65°. The braided angle is the angle between the helical wire and the axial direction of the stent. The BNS was braided by 32 strands of helical NiTi wires with a wire diameter of 0.2 mm. The BCS was braided by 8 NiTi wires and 24 PET strips. The thickness of the PET strip was 0.12mm [12]. The width of the PET strip (*w)* was 0.57 mm, which could be calculated as following:

(1) $$w=L\cos(\beta )=\frac{\pi D}{16}\;\cos(\beta )$$

where *L* is the distance of the two crossover points along the circumferential direction (Figure 1a).

The superelastic constitutive model of NiTi wires was implemented through a built-in Abaqus User Material Subroutine (VUMAT) and the material parameters were adopted from literature [13]. The PET strip was characterized by an elastoplastic material model [14]. Both material parameters were summarized in Table 1. Following the mesh-sensitivity study, each NiTi wire was meshed with 240 two-node linear beam elements (B31). The PET strips were meshed with 23,040 reduced 4-node doubly curved shell elements (S4R).

Compression tests were conducted in silico to quantify the radial strength of the stent (Figure 1c). Specifically, the middle section of the stent was vertically compressed by a rigid cylinder of 5 mm in diameter. The displacement control of 3.5 mm, *i.e.*, 50% of the outer diameter of the stent, was enforced on the cylinder [15]. The reaction force of the cylinder, *i.e.*, load applied on the stent, positively correlated with the radial strength of the stent, was monitored during the load and unload process.

The stent flexibility was quantified by the pure bending tests (Figure 1d). Both ends of the stent were fixed to a rigid plane with rotations around the X axis in opposite directions until the angle between two end surfaces reached at 60° [16]. The corresponding load, *i.e.*, bending moment, was recorded.

A general contact algorithm was considered among all contact surfaces with friction coefficients of 0.3. The kinetic energy of each stent was kept under 5% of the internal energy to avoid the inertia effect. Abaqus/Explicit solver (Dassault Systèmes Simulia Corp.) was used due to its demonstrated capacity for complex contact problems.

## 3 Results

**Figure 2:**
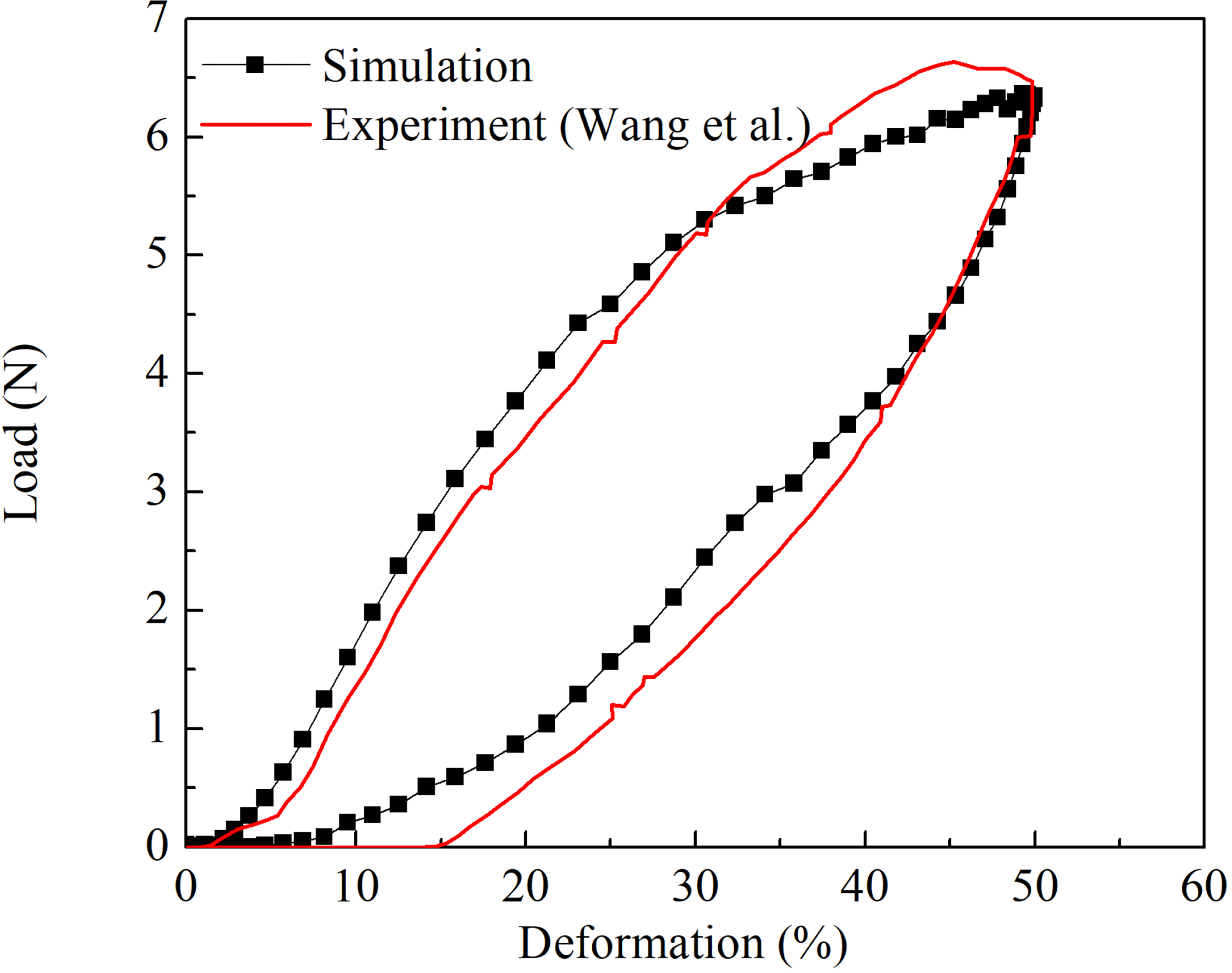
Simulation of the braided composite stent compression in compared to experimental results.

The radial strength of the BCS was compared with the published data as illustrated in Figure 2. The percentage deformation was estimated with reference to the original outer diameter of the stent. It is clear that our computational results matched well with the experiment (Model B2) [9].

**Figure 3:**
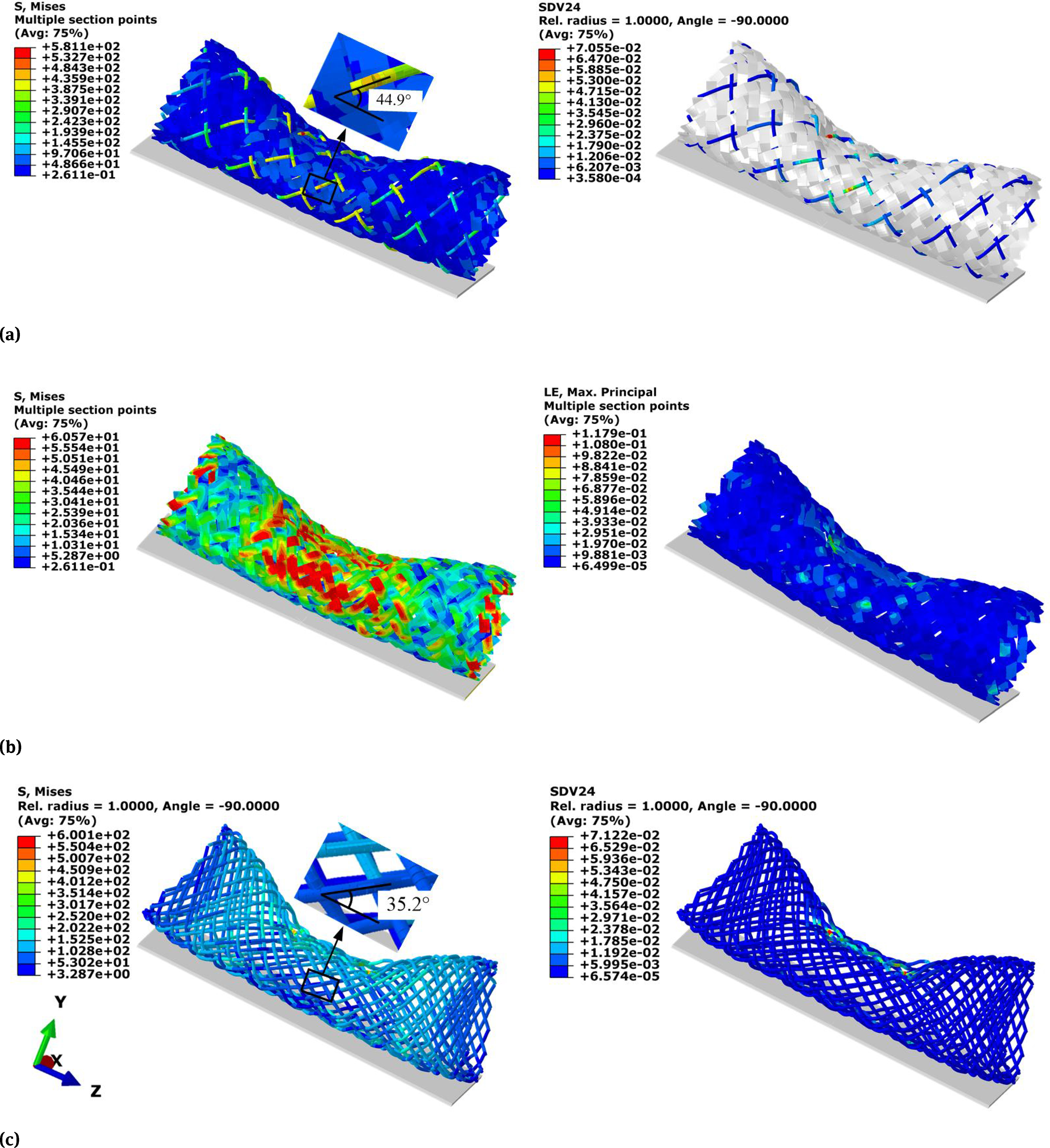
Compression induced stress (left) and strain (right) distributions for (a) nitinol wires in braided composite stent; (b) PET strips of braided composite stent; and (c) braided nitinol stents.

The compression induced stress and strain distributions in both BCS and BNS were shown in Figure 3. The peak von Mises stress occurred on the nitinol wires for both stents. It was 581MPa and 600MPa for the BCS and BNS, respectively. Both values fall in the range of transformation loading (400 to 650 MPa) as defined in Table 1. It indicated that the deformation of nitinol wires reached at the plateau of phase transformation between austenite and martensite. The maximum tensile strain (SDV24) of the nitinol wires was 7.06% in BCS and 7.12% in BNS. Both were below the elastic limit of 8%, indicating that nitinol wires could fully recover back to its original configuration following the compression. The peak von Mises stress and strain in the BCS strips was 60.6 Mpa and 11.8%, respectively. In addition, the stress or strain distribution patterns of BCS were different with the ones of BNS. Specifically, the stress and strain of the BNS concentrated beneath the cylinder surface (Figure 3c), while the stress and strain of the BCS concentrated around its lateral region (Figure 3a&b). The braiding angle at the stress concentration area of the BCS decreases from the original 65° to 44.9° while it decreases further to 35.2° for the BNS (Figure 3a & c). This indicated the wire rotation and slippage during the loading.

**Figure 4:**
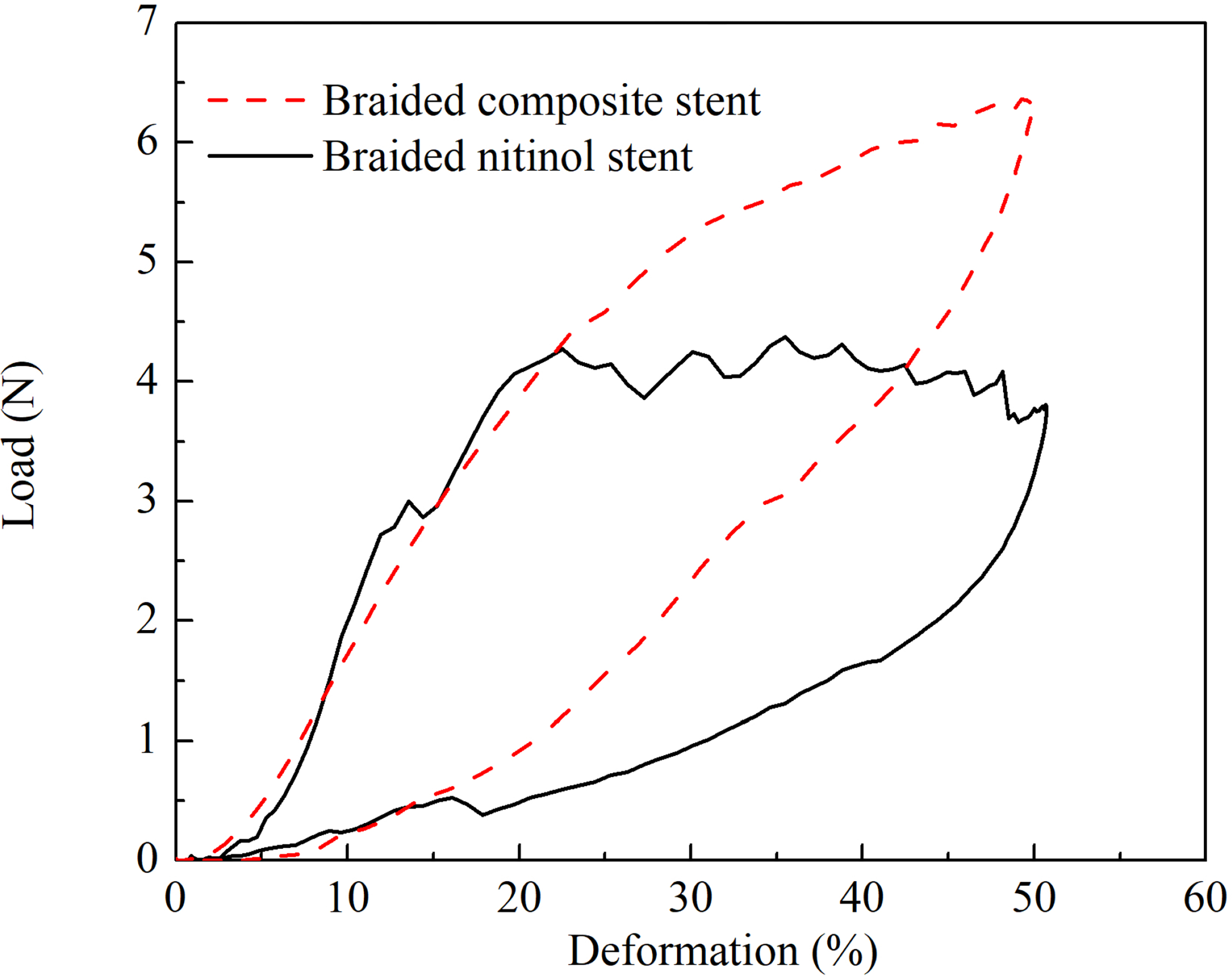
The radial compression of braided composite stent and braided nitinol stent

The load-deformation diagrams of both BCS and BNS were depicted in Figure 4. It was clear that the BCS and BNS demonstrated a similar compressive load at lower deformation, *i.e.*, less than 20%. Thereafter, the compressive load in BNS exhibited a plateau at approximately 4.2 N, while the compressive load of the BCS continually increased up to 6.4 N.

**Figure 5:**
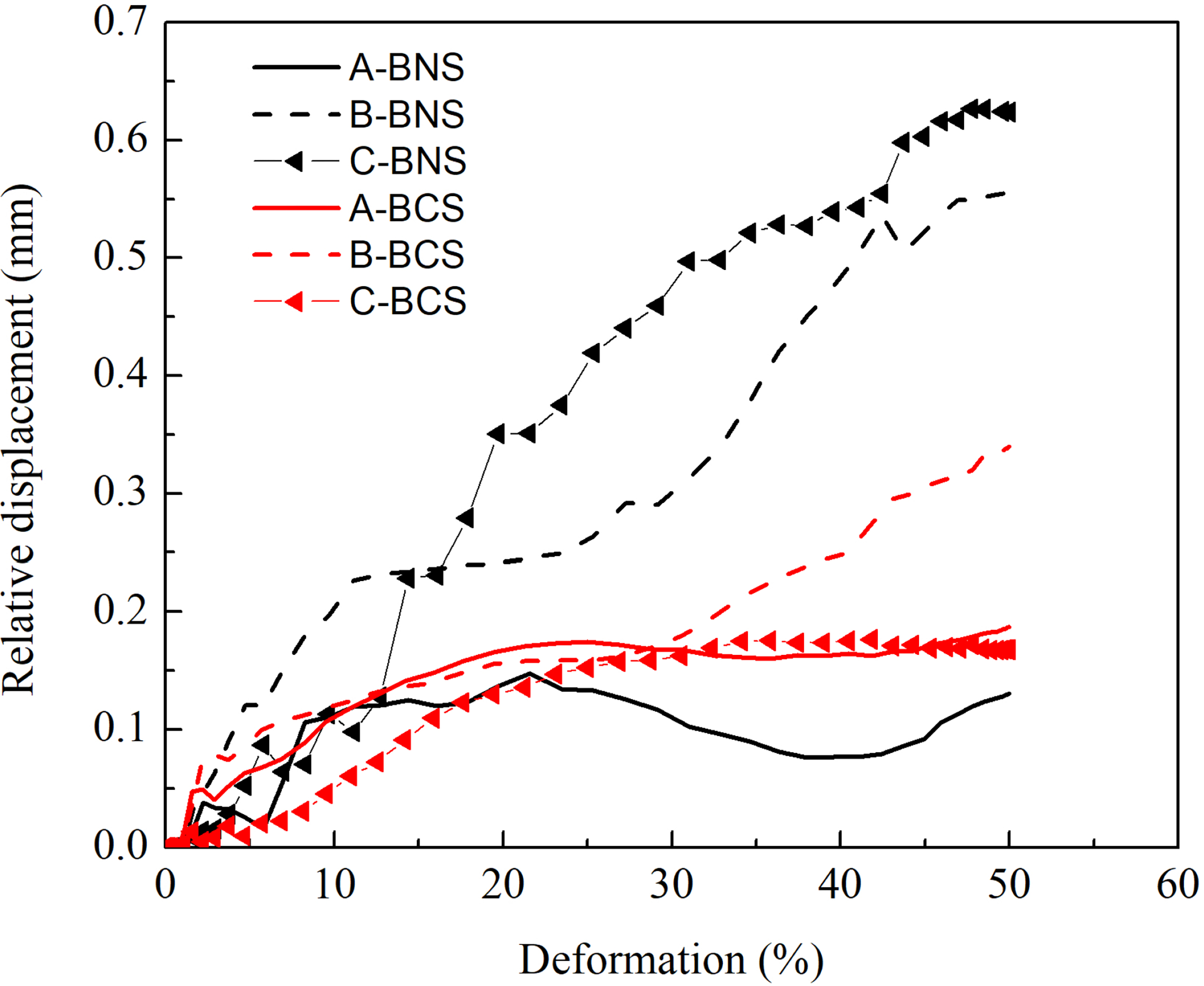
Relative displacement of two points between intersections of NITI wires of braided composite stent and braided nitinol stent under radial compression.

The slippage was estimated by monitoring the relative displacement at three representative wire intersection locations (A, B, and C, from center to the end of stent, as labeled in Figure 1). The relative displacements of these three locations subject to compression were depicted in Figure 5. It was clear that the stent deformation positively correlated with the relative deformation of wires. At the compression deformation of 50%, the relative displacements were 0.19 mm, 0.34 mm and 0.16 mm for the location A, B and C of the BCS, respectively. For the BNS, they were 0.13 mm, 0.56mm and 0.62 mm, respectively. The results showed that the BCS showed a more uniform and smaller relative displacement than the BNS, the larger relative displacement in BNS may contribute to the unstable radial force at the larger deformation.

**Figure 6:**
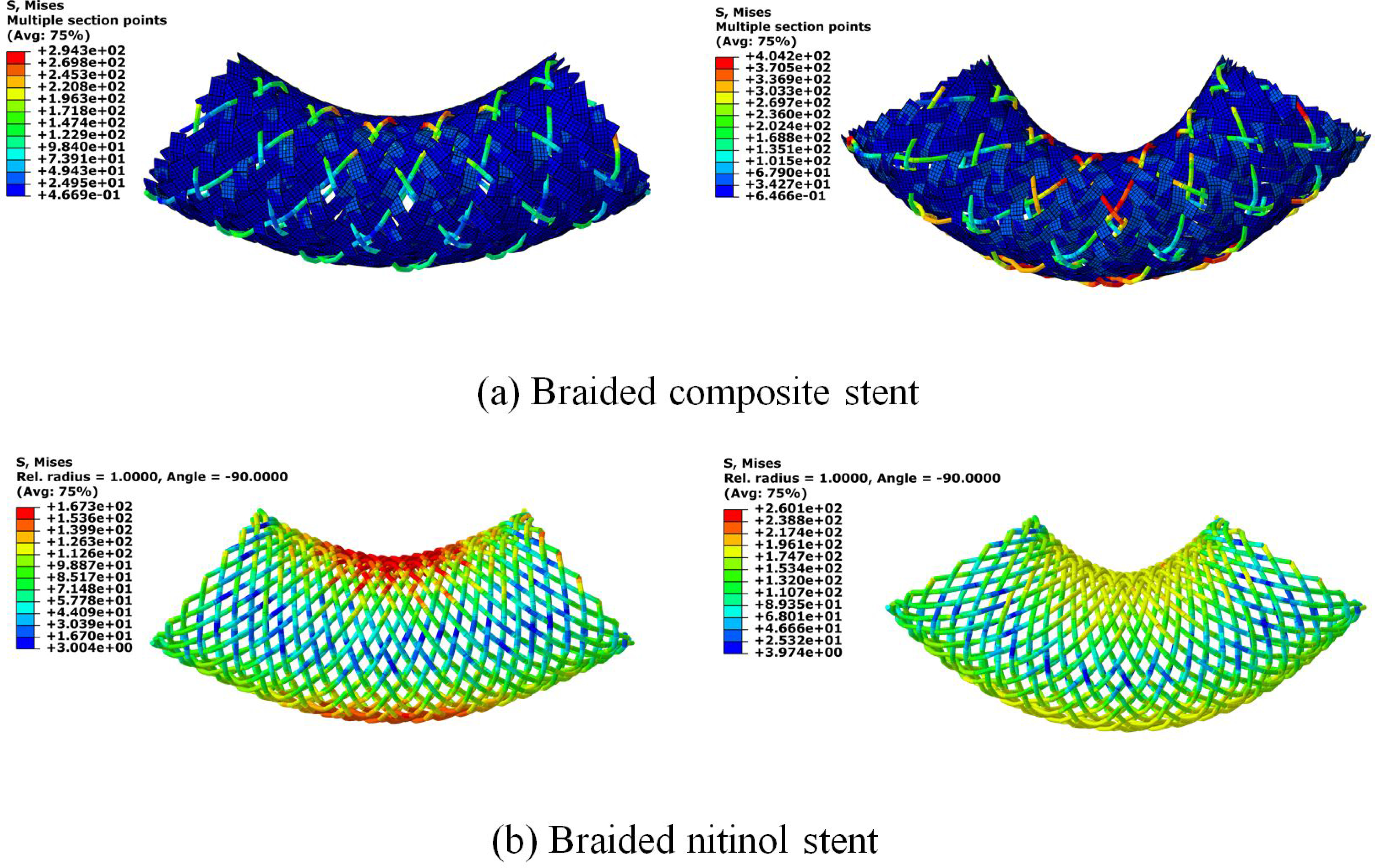
Bending behaviors of braided composite stent and braided nitinol stent at the rotation angle of 37° (left) and 60° (right).

**Figure 7:**
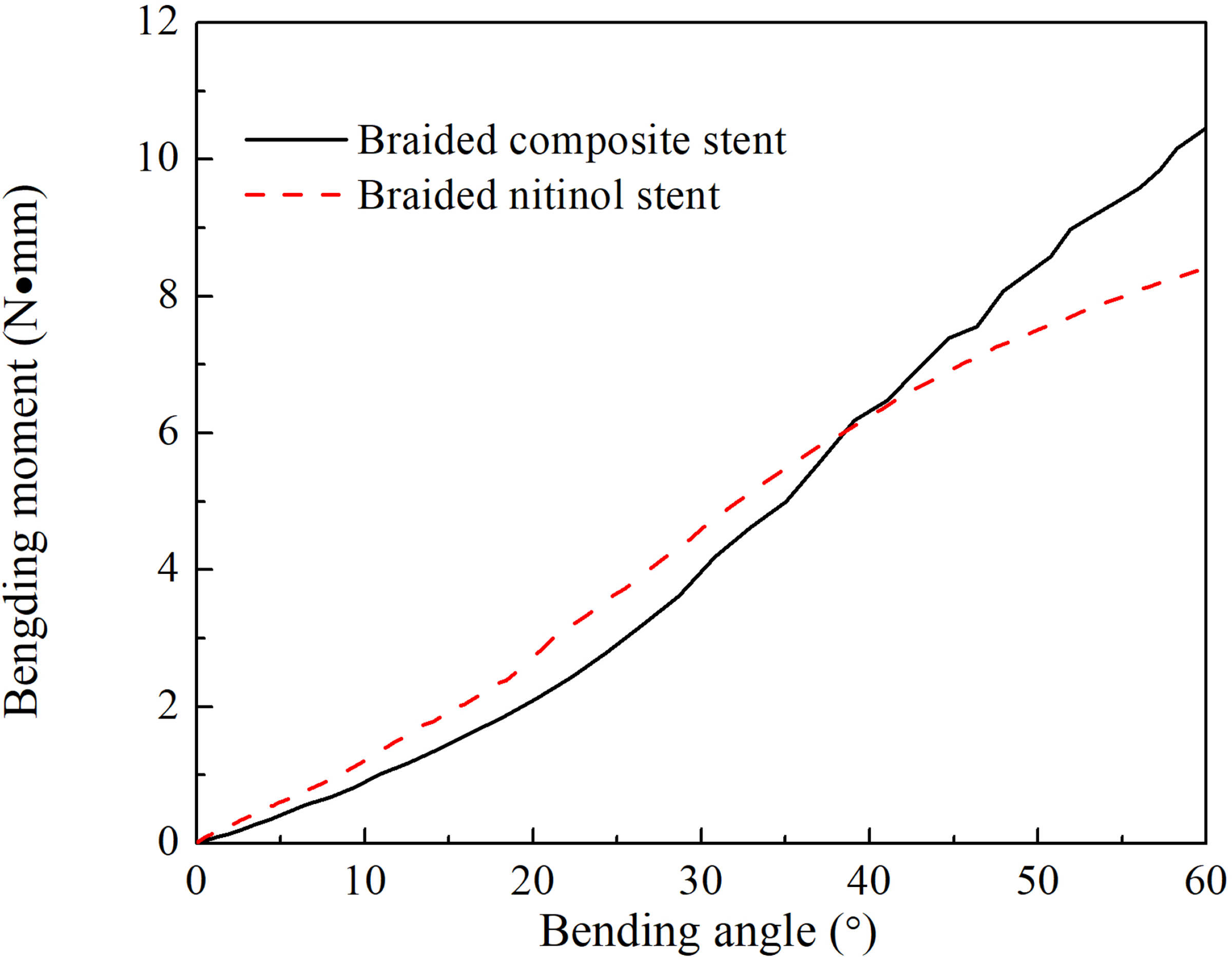
The bending behavior of braided composite stent and braided nitinol stent

The stress distribution of the stents during the bending test were shown in Figure 6. At the bending angle of 37°, the peak value of von Mises stress in BCS was 294 MPa, which was larger than that in the BNS of 167 MPa, the same trend was found at the bending angle of 60°, which was 404 MPa in BCS and 260 MPa in BNS. The peak value of von Mises stress in two stents were both located in the nitinol wires. The bending moment for each stent during the bending test was shown in Figure 7. The BCS demonstrated a relative larger bending moment than the BNS at a bending angle larger than 37°. At a bending angle of 60°, the bending moment of the BCS was 24% larger than that of the BNS.

## 4 Discussions

In this work, the design of BCS was systematically characterized in terms of radial strength and longitudinal flexibility. The mechanical behaviors of the BCS were compared with the BNS with the same weaving pattern. The major difference lies in the replacement of 24 strands of NiTi wires by the wider PET strips. The BCS possessed the advantage of covered stent without compromising their mechanical performance. The BCS provided a direct barrier to the tissue underneath. Moreover, the radial strength of the BCS was larger than the BNS especially at the larger radial deformation. The bending moment was smaller than the BNS when the bending angle was less than 37° and much less than the reported ones of the covered stent. When the polyurethane or silicone covered stents were subjected to a bending angle of 30°, the required forces were 11% and 44%, respectively, more than the BNS [17]. While at a bending angle of 30°, the bending moment of the BCS was 9.3% smaller than that of the BNS.

The radial strength of the stent, *i.e.*, the capability to scaffold the stenotic lumen, is the first consideration of the stent conceptual design [18, 19]. The BCS and BNS showed the same radial strength when the deformation is less than 20%. This seems different from the documented experiment [9], which stated that the radial strength of the BCS was much larger than the BNS. This could be explained by the different braided angle for the BCS and BNS. Specifically, it was 65° for BCS and 30° for BNS. The braided stent with a larger braided angle resulted in a higher radial strength [10]. As the deformation exceeded 20%, the radial strength of the BNS reached a plaque of 4.2 N, while the BCS kept increasing up to 6.4N. The different radial strength of the BCS and BNS at a large deformation could be attributed to the interaction between the PET strips and NiTi wires. In BNS, there existed the relative rotation and slippage between NiTi wires. However, the presence of wide PET stripes in BCS constrained the relative rotation and slippage of the NiTi wires. Our compression tests have shown that the alteration in the braided angle of the BNS was 48% larger than that of the BCS. This was also reflected in the relative larger displacement of the BNS than that of the BCS. A large relative movement in the woven wires was associate with the smaller radial strength and a larger longitudinal flexibility.

The longitudinal flexibility was required for a smooth maneuver through torturous arteries as well as implant inside a curved artery. Enhanced flexibility of stent was also associated with the decreased risk of kinking and incidence of limb thrombosis in tortuous anatomy [6, 7]. The BCS desired a larger bending moment when the bending angle was larger than 37°. This could be explained by the larger radial strength of the BCS [9] for larger deformation.

In the present work, we focused on the role of PET strips and the interaction between the PET strips and the NiTi wires on the novel BCS performance. It should be noted that this work does not completely reflect the optimal attributes of the BCS stent, which need consider more parameters, such as the mass fraction of the wires, the braided angle, diameter of the NiTi wires, strip thickness and material properties, etc. The interaction between stent and a torturous artery could provide more complete information on the assessment of this novel stent design. Despite these limitations, the present work demonstrated the potential of the BCS as a substitute for a covered stent with improved mechanical behaviors, which might have significant clinical implications for in-stent restenosis [20].

## Figures and Tables

**Table 1:** Material properties of nitinol and polyethylene terephthalate.

Nitinol	
Austenite elasticity *E_A_* (MPa)	50000
Martensite elasticity *E_M_* (MPa)	37000
Start of transformation loading *σ*S L (MPa)	400
End of transformation loading *σ*E L (MPa)	650
Start of transformation unloading *σ*S U (MPa)	350
End of transformation unloading *σ*E U (MPa)	80
Volumetric transformation strain *ϵ*LV	0.055

Polyethylene terephthalate (PET)	
Young’s modulus E (GPa)	3.5
Poisson ratio *υ*	0.4
Yield stress *σ_s_* (MPa)	60
Tensile stress *σ_t_* (MPa)	70
